# Lipidomics Unravels the Role of Leaf Lipids in Thyme Plant Response to Drought Stress

**DOI:** 10.3390/ijms18102067

**Published:** 2017-09-28

**Authors:** Parviz Moradi, Atiyeh Mahdavi, Maryam Khoshkam, Marcello Iriti

**Affiliations:** 1 Research Division of Natural Resources, Zanjan Agricultural and Natural Resources Research and Education Centre, AREEO, Zanjan 45331-47183, Iran; 2 Institute for Advanced Studies in Basic Sciences (IASBS), Zanjan 45137-66731, Iran; 3 Department of Chemistry, University of Mohaghegh Ardabili, Ardabil 56199-11367, Iran; khoshkam@uma.ac.ir; 4 Department of Agricultural and Environmental Sciences, Milan State University, 20133 Milan, Italy

**Keywords:** lipidome, FT-ICR, water deficit, *Thymus*, stress physiology, lipid signaling

## Abstract

*Thymus* is one of the best known genera within the Labiatae (Lamiaceae) family, with more than 200 species and many medicinal and culinary uses. The effects of prolonged drought on lipid profile were investigated in tolerant and sensitive thyme plants (*Thymus serpyllum* L. and *Thymus vulgaris* L., respectively). Non-targeted non-polar metabolite profiling was carried out using Fourier transform ion cyclotron resonance (FT-ICR) mass spectrometry with one-month-old plants exposed to drought stress, and their morpho-physiological parameters were also evaluated. Tolerant and sensitive plants exhibited clearly different responses at a physiological level. In addition, different trends for a number of non-polar metabolites were observed when comparing stressed and control samples, for both sensitive and tolerant plants. Sensitive plants showed the highest decrease (55%) in main lipid components such as galactolipids and phospholipids. In tolerant plants, the level of lipids involved in signaling increased, while intensities of those induced by stress (e.g., oxylipins) dramatically decreased (50–60%), in particular with respect to metabolites with *m*/*z* values of 519.3331, 521.3488, and 581.3709. Partial least square discriminant analysis separated all the samples into four groups: tolerant watered, tolerant stressed, sensitive watered and sensitive stressed. The combination of lipid profiling and physiological parameters represented a promising tool for investigating the mechanisms of plant response to drought stress at non-polar metabolome level.

## 1. Introduction

In their environment, plants are often exposed to a plethora of biotic and abiotic stresses, such as drought, salinity, extreme temperatures, nutritional deficiencies, heavy metals, and pollutants, as well as pathogen and insect attacks [1,2,3]. These factors reduce crop yield and therefore, cause global economic losses [4]. It has been estimated that drought, a major type of abiotic stress, affects around 64% of the world’s land area [5,6]. In plants, water is involved in a number of pivotal physiological functions, such as plant growth and photosynthesis, and water deficiency results in a severe and often lethal stress in plant cells. Water deficit is characterized by morphological and physiological features in plants, such as decreased leaf water potential, wilting, stomatal closure, reduced gas exchange and photosynthesis, and, finally, plant death [7,8]. Therefore, drought stress may largely impact crop yield and quality and, hence, food security [9,10].

At a metabolic level, sensitive plants usually exhibit a higher number ofsignificantly altered metabolites than tolerant plants, though, qualitatively, the latter show increases in the accumulation of osmolytes (that maintain the cell water status), and antioxidants that protect plant cells from reactive oxygen species (ROS) produced during drought stress [1,2,3]. Lipidome changes and membrane lipid remodelling are relevant strategies adopted by plant cells to counteract abiotic stresses such as drought.

Lipids are cell macromolecules with essential structural, energy storage and signaling roles in biological systems [11]. Of note, lipids possess two major roles in the organism’s response to stress. First, they act as signaling mediators [12,13]; second, they play an important role in the process of alleviating the deleterious effects of stress [14,15]. Different groups of lipids are involved in signaling systems, including lysophospholipids, fatty acids, phosphatidic acid, inositol phosphate, diacylglycerols, oxylipins, sphingolipids, and *N*-acylethanolamine [12,13,16,17,18]. Signaling lipids are quickly synthesized upon stress initiation by a wide range of enzymes including phospholipases, acyl hydrolases, phytosphingosine kinases, diacylglcerol kinases, and fatty acid amide hydrolases [12,13,19]. Moreover, lipids mitigate cell damage through membrane remodeling in response to abiotic stresses [14,20,21]. Such remodeling, finally, maintains lipid dynamics and membrane protein functionality [22].

Thyme belongs to the *Thymus* genus and the Lamiaceae (Labiatae) family, one of the largest families of dicotyledons rich in aromatic plant species [23]. Thyme itself, a perennial herb, has been well-known worldwide since ancient times for its medicinal and culinary uses, and its extracts possess antiseptic, antibacterial and spasmolytic properties [24,25]. Rapidly increasing demand for various thyme products indicates the importance of further research on this plant, in order to comprehend the mechanisms involved in plant adaptation to different abiotic/biotic stresses [4].

Considering the economic and phytotherapeutic importance of this genus, investigation on the impact of environmental stresses, such as water deficit, could be very informative and useful. Indeed, this information would undoubtedly assist plant producers and breeders to gain more yield under stressed conditions. In this regard, we recently investigated the response of *Thymus* spp. populations to drought stress [26]. Indeed, in a previous study, we screened different thyme populations based on their morpho-physiological traits and tolerance to drought stress. The main aim of this study is to provide a comprehensive overview of the lipidome changes in thyme plants under severe drought stress, and to elucidate mechanisms involved in plant adaptation and tolerance to water deficiency. Experiments were carried out on thyme species tolerant or sensitive to drought (*Thymus serpyllum* L. and *Thymus vulgaris* L., respectively), and their lipid profiles were compared under stress conditions.

## 2. Results and Discussion

### 2.1. Lipid Profile Changes in Sensitive Thyme Plants under Drought Conditions

**Figure 1:**
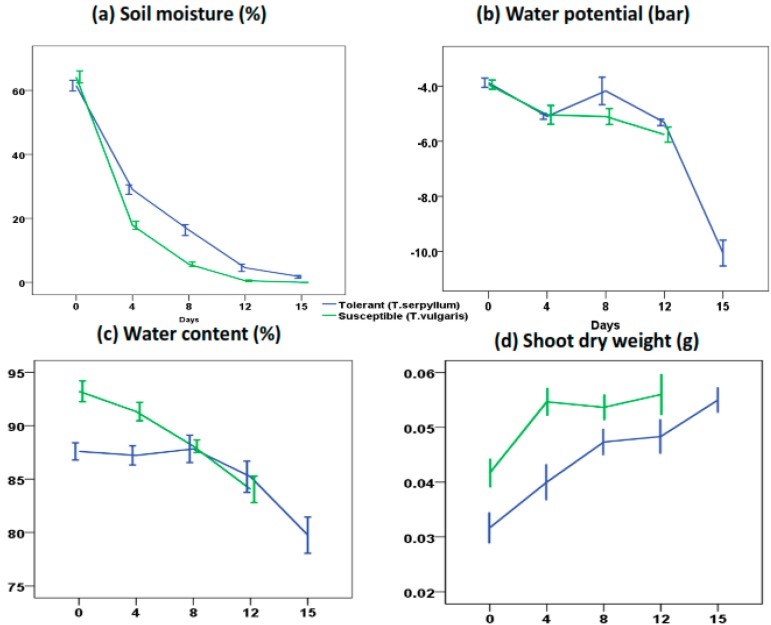
Impact of drought stress on physiological parameters in tolerant and sensitive thyme [15]: (**a**) soil moisture (%); (**b**) water potential (bar); (**c**) water content (%); and (**d**) shoot dry weight (g). Drought stress was imposed by water withholding on one-month-old plants of tolerant and sensitive populations (*Thymus* *serpyllum* and *Thymus* *vulgaris*, respectively). Physiological parameters were recorded at 4-day intervals.

Prior to lipid profiling, physiological parameters (soil moisture, water potential, water content and shoot dry weight) of plants under conditions of water deficiency were recorded to indicate how drought stress progresses (Figure 1) [15]. To investigate the effects of water deficit on sensitive thyme plants, non-polar metabolites were profiled. In this experiment, 2527 non-polar metabolites were detected in negative mode, though only 84 metabolites significantly changed. The complete list of the identified metabolites that significantly changed in sensitive plants under drought is reported in Table S1.

**Figure 2:**
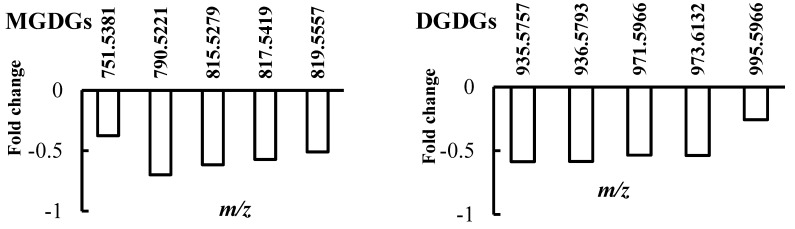

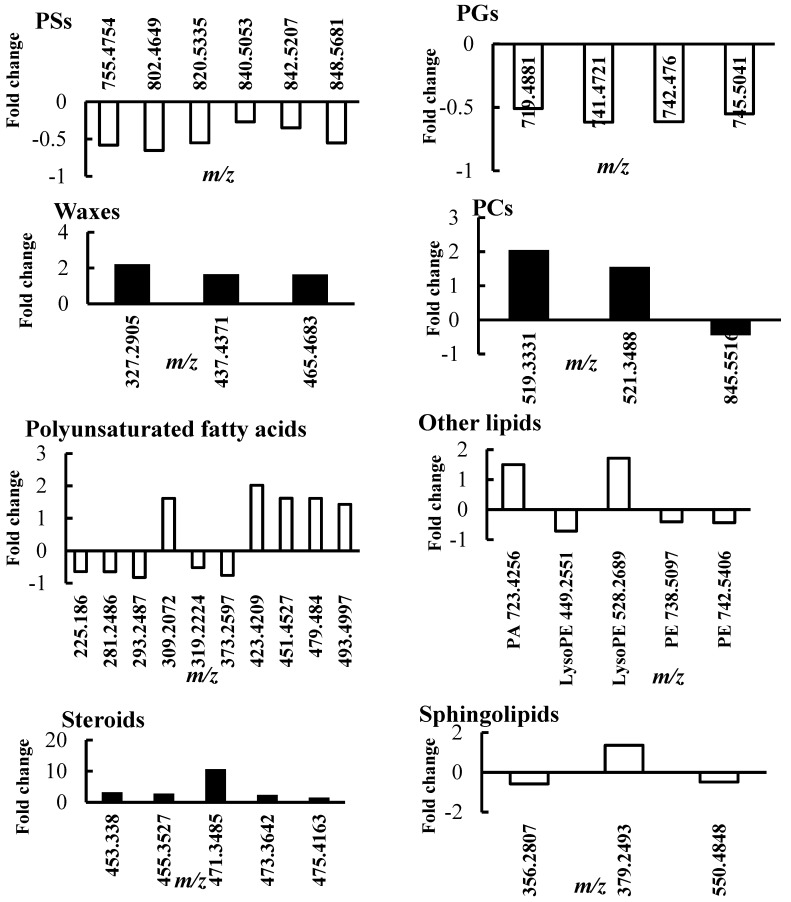
Lipid composition changes in sensitive thyme plants under drought conditions. After water withholding, thyme leaves were harvested and non-polar extracts analyzed by direct infusion Fourier transform ion cyclotron resonance (DI FT-ICR) mass spectrometry. Vertical axis represents the fold change between the control and stressed plants. The mass-to-charge (*m*/*z*) values are reported next to each bar (abbreviations: MGDGs, monogalactosyldiacylglycerols; DGDGs, digalactosyldiacylglycerols; PS, phosphatidylserine; PG, phosphatidylglycerol; PC, phosphatidylcholine).

The comparison of non-polar metabolite intensities in sensitive plants under watered and drought conditions is shown in Figure 2. Water deficiency strongly altered lipid composition. The major lipid class levels significantly decreased under drought stress, as well as total lipid levels. This trend was more evident for the main lipid components such as galactolipids (monogalactosyldiacylglycerols (MGDGs) and digalactosyldiacylglycerols (DGDGs)) as well as phospholipids (phosphatidylglycerol (PG), Phosphatidylethanolamine (PE), Phosphatidic acid (PA) and phosphatidylserine (PS)) which decreased by 55%. Among MGDGs, the metabolite with an *m*/*z* value of 790.5221 was the most affected, decreasing by 70% in the stressed plants. Though some studies reported an increase of PG in rapeseed seedlings [9], our results are consistent with those obtained in rapeseed leaves [10,11], wheat [12] and *Lupinus albus* L. genotypes [13]. Furthermore, some authors documented unchanged levels for DGDGs after water deficit [12].

Since galactolipids are mainly involved in the structure of stroma lamellae, the grana system, and the chloroplast envelope [14], water deficit would damage reaction centres and, consequently, inhibit photosynthesis [15]. Decreased galactolipid levels have been pointed out in previous investigations as a main trait of sensitive plants under drought stress [12,13,16,17]. In turn, MGDG reduction could result from higher activity of MGDG-hydrolases under drought stress conditions [18].

A contrasting trend emerged for steroids, fatty acids and some sphingolipids which increased in stressed plants compared to the watered ones (Figure 2). Regarding sphingolpids, sphinganine 1-phosphate levels increased by 136%, while the others exhibited a mean decrease of 53%. Free fatty acids (FFAs) were the major lipid constituents in the sensitive thyme leaves. As shown in Figure 1, FFAs exhibited different trends following water deficit, varying from a 52% decrease in *cis*-2-octadecenoate intensities to a 200% increase in octacosanoic acid.

In addition, the large increase in lipid unsaturation observed in this experiment caused a reduced ratio of the saturated lipids versus unsaturated lipids. Even though this result is in contrast with those obtained in other plants such as cotton [19], rapeseed [10], and safflower [20] under drought stress, it should be noted that in previous comparative studies including two plant species with diverse tolerance level, a correlation was observed between lipid unsaturation and stress tolerance [21,22,23]. Therefore, increased levels of unsaturated lipids may occur in tolerant plants.

On the other hand, the DGDG/MGDG ratio, another important indicator of plant tolerance to stress, was almost unchanged following water depletion in sensitive plants, at 0.93 and 0.97 under watered and drought conditions, respectively.

### 2.2. Lipid Profile Changes in Tolerant Thyme Plants under Drought Conditions

**Figure 3:**
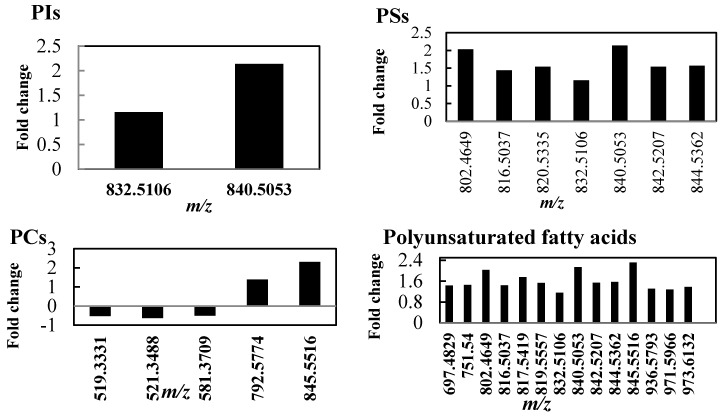

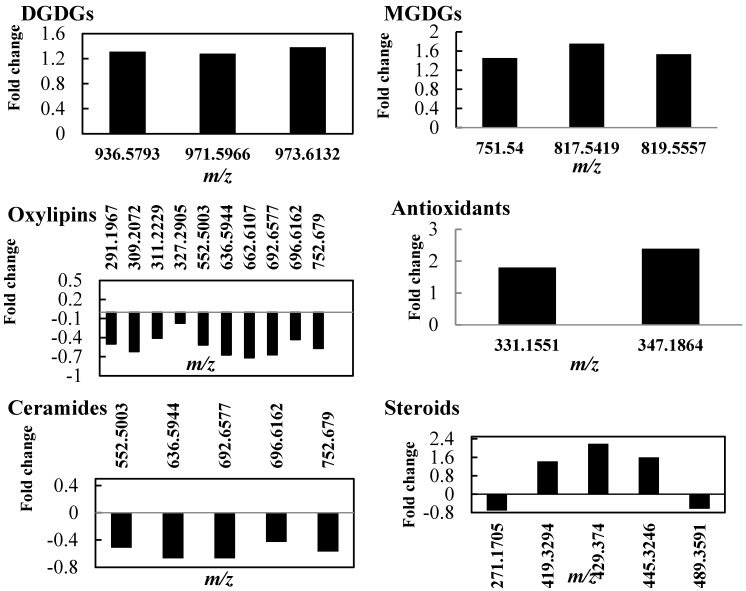
Lipid composition changes in tolerant thyme plants under drought conditions. After withholding water, thyme leaves were harvested and non-polar extracts were analyzed by DI FT-ICR mass spectrometry. The vertical axis represents the fold change between the control and stressed plants. The mass-to-charge (*m/z*) values are reported next to each bar (abbreviations: PI, phosphatidylinositol; PS, phosphatidylserine; PC, phosphatidylcholin; DGDGs, digalactosyldiacylglycerols; MGDGs, monogalactosyldiacylglycerols).

A full list of identified non-polar metabolites significantly changed in tolerant thyme plants under water deficit condition is reported in Table S2. Changes in the intensities of lipids in plants under watered and drought conditions are shown in Figure 3. Though the levels of major lipids increased in stressed plants, indicating an increase in lipid biosynthesis and/or a decrease in lipid degradation, a dramatic decrease was observed in the intensities of stress-induced lipids (oxylipins). Among the detected phospholipids, including phosphatidylinositol (PI), PS, and phosphatidylcholine (PC), the metabolites with *m*/*z* values of 519.3331, 521.3488 and 581.3709 decreased by 50–60%, whereas those with *m*/*z* values of 845.5516, 840.5053, and 840.5053 were the most affected phospholipids, increasing by over 200% in response to drought stress. The amounts of other phospholipids were also increased in the stressed plants as compared with the watered controls.

Increase of fatty acid unsaturation has been considered as one of the most striking features of drought-tolerant plants, scavenging ROS and reducing damage to cell lipids [23,24]. Analysis of the unsaturated fatty acid composition showed increased levels of all detected polyunsaturated fatty acids (PUFAs) 18:2 and 18:3 (Figure 3) in the drought-stressed thyme plants. A similar pattern was also reported for *Arabidopsis thaliana* [23]. Furthermore, PUFAs with *m*/*z* values of 802.4649, 840.5053 and 845.5516 showed the highest increase compared to other unsaturated fatty acids.

Another feature of tolerance to drought stress is an increased digalactosyldiacylglycerol (DGDG):monogalactosyldiacylglycerol (MGDG) ratio [24]. Our results showed a general increase in both membrane structural lipids DGDGs and MGDGs in the stressed leaves, though a slight decrease in DGDG/MGDG ratio was registered in these plants as compared to watered controls (Figure 3). A recent study also reported increased levels of all structural lipids of the photosynthetic membranes (DGDGs, MGDGs, and sulfoquinovosyldiacylglycerols) in the highly drought-tolerant plant *Calotropis procera* W. T. Aiton [25].

Analysis of the changes in oxylipin profile revealed that the content of the oxidized fatty acids was significantly decreased in the stressed thyme plants (Figure 3). Since these fatty acids are mainly produced by the action of ROS [24,26], it can be suggested that the tolerant thyme plants efficiently scavenge ROS arising from drought stress, thus reducing non-enzymatic formation of oxylipins. Interestingly, the content of antioxidant compounds significantly increased in leaves of stressed thyme plants (Figure 3) [27]. These antioxidants reduce ROS fatty acid oxidation and oxylipin production, thus protecting cells against oxidative damage.

Other changes in the lipid profile caused by drought stress included a decrease in the levels of ceramides, while levels of steroids increased (Figure 3). According to previous studies, the stress-induced changes in the profile of mentioned lipid classes can cause membrane lipid remodeling and activate plant defense responses against various biotic and abiotic stresses such as drought [26,28].

### 2.3. Integrative Analysis of Lipid Composition in Drought-Sensitive and -Tolerant Thyme Plants

#### 2.3.1. Principal Component Analysis and Pattern Recognition Analysis of Mass Spectra

Principal component analysis (PCA) was performed on the pretreated data and score plots were constructed (Figure S1).

**Figure 4:**
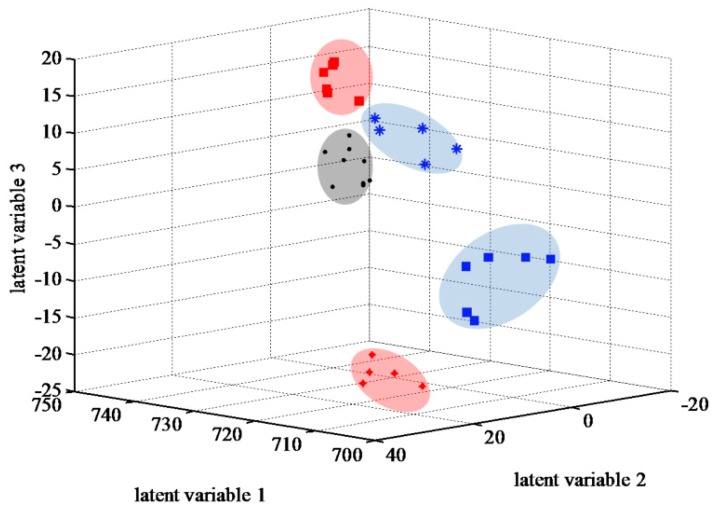
Score plot obtained by partial least square discriminant analysis (PLSDA). Four distinct groups of samples were separated: ● quality control (QC); ★ tolerant watered (TW); ▄ tolerant drought-stressed (TD); ★ sensitive watered (SW); ▄ sensitive drought-stressed (SD).

Because of partial overlap between tolerant watered (TW) and tolerant drought-stressed (TD) groups, partial least square discriminant analysis (PLSDA), a supervised pattern recognition method, was applied to maximize the separation between the different groups (Figure S1) [29,30,31]. This technique is useful for data with a much higher number of variables than samples and with multicolinearity in the dataset [29], and provides several statistics such as loading weight, variable importance on projection (VIP), and the regression coefficient, that can be used in metabolite identification. This technique provides a visual interpretation of a large dataset through a low-dimensional and easily interpretable score plot (Figure 4) [31]. Of note, TW and TD groups were completely separated and stress-induced changes in various metabolites were associated with water deficiency. The PLSDA visualized individual groups and indicated appropriate reproducibility of the FT-ICR-metabolomics based on the trend of grouped samples.

#### 2.3.2. Outstanding Metabolites Involved in Plant Response Mechanism

**Figure 5:**
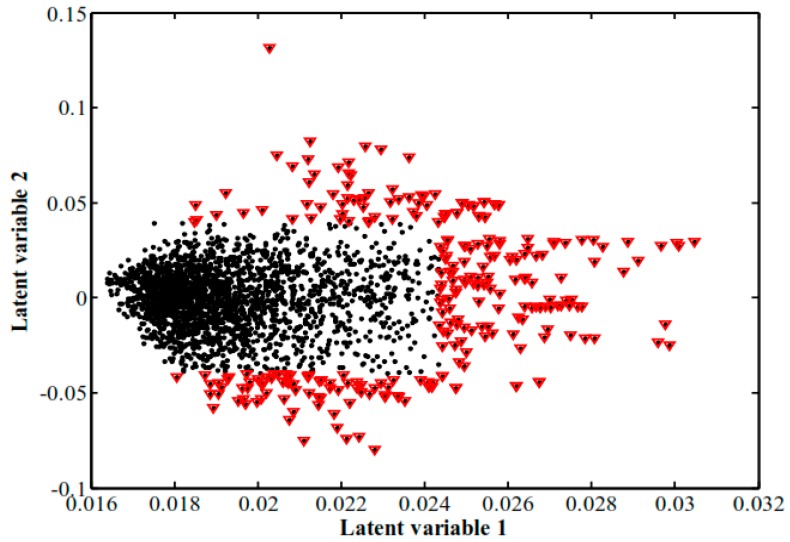
The most significant peaks based on high and low scores identified on the partial least square discriminant analysis (PLSDA) loading plot. In order to determine the most important metabolites, 142 peaks were selected (red triangles). These peaks showed the greatest influence on the classification of samples, as they were placed in the lowest and highest values of latent variables 1 and 2. The cut off line was calculated based on the formula: $V-\bar{V}\geq \alpha \times \sigma$.

PLSDA loading plots were then constructed to identify metabolites responsible for sample separation (Figure 5).The selected points are represented as red triangles in Figure 5.

**Figure 6:**
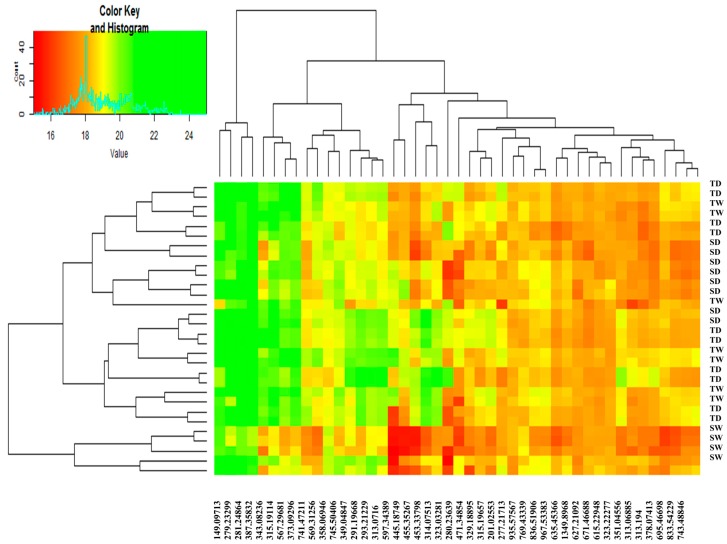
Hierarchically clustered heat map of the data. Forty-five peaks were divided into four groups (SD, sensitive drought-stressed; SW, sensitive watered; TD, tolerant drought-stressed; TW: tolerant watered). Different colors indicate the relative abundance of each metabolite in different conditions.

Heat maps were used for data visualization [32,33,34]. Based on the heat map analysis, all metabolites in the SD and TD groups were affected by drought stress, with a significant difference in their changing patterns (Figure 6). Metabolites with *m*/*z* values of 149.07713, 279.23299, 281.24864, and 367.35832 showed the highest intensities in all four groups and were not affected by water deficit in either sensitive and tolerant plants. Consequently, these metabolites did not seem to be involved in the stress response mechanisms of thyme plants. While intensities of most metabolites changed following stress (both in tolerant and sensitive groups) as compared to the corresponding watered plants, dramatic changes in concentrations of some metabolites were recorded. For instance, the intensities of the *m*/*z* values 323.03281 and 323.22277 significantly increased in the tolerant plants under drought conditions as compared to the control ones. In contrast, metabolites with *m*/*z* values of 597.34385 and 314.07513 decreased in the stressed plants as compared to the watered groups. Other stress-induced changes in metabolite intensities were documented in sensitive thyme plants, i.e., an increase of *m*/*z* values 597.34384, and 323.03281 and decrease of *m*/*z* values 343.08236 and 291.19366 in stressed plants as compared to the controls.

### 2.4. Metabolic Pathway Analysis

Selected important metabolites were introduced into MetaboAnalyst online database (http://www.metaboanalyst.ca) and MI-Pack software, and the all obtained pathways are reported in Table S3. Among these pathways, those with *p* < 0.05 from MetaboAnalyst and with coverage value ≥0.1 from MI-Pack were selected and are shown in Table 1.

The α-linolenic acid (18:3) metabolismwas significantly involved in drought stress response of thyme plants; indeed, α-linolenic acid represents one of the main components of the plant cell membrane [35]. Of note, this PUFA is also relevant for biosynthesis of jasmonic acid and oxylipinin higher plants via the phospholipase/lipoxygenasepathway [36]. The increase of flavonoids and isoflavonoids in stressedplantsis in agreement with recent reports on other plant species [37,38]. Indeed, these secondary metabolitescan scavenge ROS and protect plant cellfromoxidative stress caused by water deficiency [39,40]. The role of biosynthesis of steroids and other unsaturated fatty acidsin drought stressresponse of thyme plantshas been previouslydiscussed.

**Figure 7:**
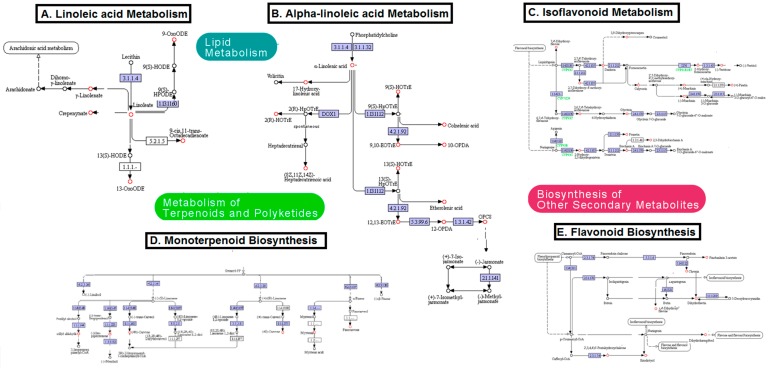
Major metabolic pathways significantly involved in drought stress response of thyme plants; significantly changed metabolites are indicated with a red circle. All pathways were obtained from MetaboAnalyst online database (available online: http://www.metaboanalyst.ca) and MI-Pack software (hi-resolution image is presented in Figure S2).

In the linoleic acid metabolic pathway, (10*E*,12*Z*)-9-oxooctadeca-10,12-dienoic acid (9-oxoODE), γ-linolenate, linolenate, cepenynate, 13-oxooctadeca-9,11-dienoic acid (13-oxoODE), and 9-*cis*,11-*trans*-octadecadinoate changed significantly following drought stress (Figure 7A, red circles). In the α-linolenic acid pathway, significantly altered metabolites included α-linolenic acid, 17-hydroxy linolenic acid, (9*S*,10*E*,12*Z*,15*Z*)-9-hydroxyoctadeca-10,12,15-trienoic acid (9*S*-HOTrE), 9, 10 EOTrE, colnelenic acid, 8-[(1*S*,5*S*)-2-oxo-5-[(*Z*)-pent-2-enyl]cyclopent-3-en-1-yl]octanoic acid (10-OPDA), 2(*R*)-HOTrE, etherolenic acid, 12-oxo-*cis*-10, *cis*-15-phytodienoic acid (12 OPDA), and 8-[3-oxo-2-[(Z)-pent-2-enyl]cyclopentyl]octanoate (OPC8), 12,13-EOTrE (Figure 7B, red circles). In the isoflavonoid biosynthetic pathway, 7,4-dihydroxy-flavone, calycosin, vestitone, 2-hydroxy-formonentin, pisatin, maackiain, 2,6,7,4-tetrahydroxy-isoflavanone, glycitein, 2-hydroxy-2,3-dihydrogenistein, prunetin, biochanin, and 2,3-dihydrobiochanin A significantly changed following water deficiency (Figure 7C, red circles). The monoterpenoid metabolic route showed significantly altered levels of *S*-limonene, *R*-limonene, α-pinene, pinocarvone, myrtenal, 4*S*-carvone, 4*R*-carvone, iso-pipenitenone, and perillyl aldehyde (Figure 7D, red circles). Finally, subsequent to water deficit, flavonoid biosynthesis also showed significant changes in pinobanksin 3-acetate, chrysin, dihydrofisetin, 7,4-dihydroxy flavone, dihydrokaempferol, pentahydroxychalcone and eriodictyol (Figure 7E, red circles).

## 3. Materials and Methods

### 3.1. Chemicals, Plant Materials, and Physiological Parameters

Chemicals were obtained from Sigma (St. Louis, MO, USA). Thyme plant seeds were purchased from SemillasSilvestres^®^, Spain. *Thymus vulgaris* L. and *Thymus serpyllum* L. were used as sensitive and tolerant plants, respectively. A growth room with a 16:8 light:dark cycle and a temperature of 22 ± 0.5 °C was used to grow plants watered weekly. Experiments were carried out in triplicate. Drought stress was imposed by ceasing the watering on day 30, as described previously [26]. Briefly, seeds were sown in pots measuring 8 cm in diameter, and grown in a room with a 16:8 light:dark cycle and a temperature of 22 °C; they were watered weekly. The soil mixture was four parts Humax Multipurpose peat based compost mixed in one part perlite with Intercept 70wg insecticide added to 0.02g/L compost. To measure the soil moisture, a sensor model SM300 by Delta-T Devices Ltd was used. The SM300 measures volumetric soil moisture content with 2.5% accuracy. A pressure chamber (Skye Company Model SKPM 1400, Powys, UK) was used at midday to measure shoot water potential in shoots measuring 10–30 mm in length. To record the water potential (before and after drought), stems were cut and sealed into the chamber.

### 3.2. Tissue Harvesting and Sample Extraction

Leaf collection was performed at the end of stress period (day 12 for sensitive plants and day 15 for tolerant plants) for lipidomics experiments. Leaves at the same developmental stage were cut with scissors, flash-frozen with liquid nitrogen, and stored at −70 °C. Six biological replicates for each group were freeze-dried. To extract non-polar metabolites, the methanol: chloroform: water protocol was used. In brief, 32 μL MeOH and 12.8 μL water were added per mg of tissue, and a Precellys 24 homogenizer (Bertin Technologies Ltd, Paris, France) was used to homogenize tissues. Then, 32 μL of chloroform (CHCl_3_) and 16 μL of water were added and the mixture was centrifuged in glass vials at 4000 rpm, at 4 °C for 10 min. Finally, non-polar extracts from the lower layer in the biphasic system were transferred to vials, dried by nitrogen gas and stored at −70 °C until analysis (for more details refer to [27]).

### 3.3. Lipidome Profiling by Direct Infusion Fourier Transform Ion Cyclotron Resonance (DI FT-ICR) Mass Spectrometry

MeOH:H2O (HPLC grade) was used in an 80:20 (*v*/*v*) ratio to re-suspend freeze dried samples and then, 20 mM ammonium acetate were added (0.25% of total volume) at dilution ratio of 3:1 (dilution solvent: original extract volume). The diluted samples were vortexed and sonicated for 5 min. Randomly selected samples were mixed in an equal volume to prepare quality control (QC). All samples and QCs were centrifuged at 14,000 rpm, at 4 °C for 10 min. For each sample, 10-μL aliquots were loaded into auto-sampler plates as three technical replicates. To analyze the samples, a hybrid 7-T Fourier-transformed ion cyclotron resonance mass spectrometer (LTQ FT, Thermo Scientific, Bremen, Germany) equipped with a chip-based direct infusion nanoelectrospray ionization assembly (Triversa, Advion Biosciences, Ithaca, NY, USA) was used. To control the nanoelectrospray conditions, ChipSoft software (version 8.1.0, Advion Biosciences) was used with a 200 nL/min flow rate, 0.3 psi backing pressure and −1.7 kV electrospray voltage for negative ions. Scanning took 2 min and 15 s in total, in seven overlapping Selected Ion Monitoring (SIM) scans with range of 70–2000 *m*/*z*.

Data analysis was carried out in three stages, including pre-processing, metabolite identification and statistical analysis. In pre-processing, raw data were exported from Xcalibar (Version 2.0.7, Thermo Scientific) to MATLAB version 7 (The Mathwork Inc., Natick, MA, USA) and then subjected to custom-written code including the sum of transient files and their process [28]. The processing method was carried out as described previously [29]. Briefly, two out of three mass spectra for technical replicates with an 80% sample filter were retained. Then, the custom-written code including the sum of transient files and their processing was applied to raw data [28]. The processed transient data files were subjected to the SIM-stitch algorithm version 2.8 (custom written codes in MATLAB) as well as three more MATLAB scripts (peak filtering) [30]. At this stage, a peak list and a peak matrix were generated. The peak list contained mass to charge (*m*/*z*) signals and related intensities. The peak matrix consisted of a multivariate dataset that recorded all the peaks detected for each biological replicate.

### 3.4. Identification of the Extracted Metabolites

To identify the metabolites, the peak lists and intensities were submitted to the MI-Pack software package [31]. Empirical formulas were generated for each given accurate mass using seven “golden rules” [32]. These rules have been described in detail in our previous study [29]. The elemental composition of the detected peaks corresponding to the adducts of neutral metabolites (charged molecular ions) was added by adduct mass of the seven most relevant positive ions including M − e^+^, M + Na^+^, M + H^+^, M + 2Na-H^+^, M + ^39^K^+^, M + 2^39^K-H^+^, M + ^41^K^+^. All possible formulas were finally filtered for selection of the most accurate and correct elemental formula using the given rules.

Of note, despite the high mass accuracy, one mass might be assigned to different elemental formulas, or even similar formulas but with different structure. Therefore, all possible compounds were reported in the tables of the present study. For instance, for the *m*/*z* = 815.5279 value, all forms of 18:1–18:3-MGDG (monogalactosyldiacylglycerol) and 18:2–18:3-MGDG were considered and FTMS cannot distinguish between these isomers.

In present study, after selecting *m*/*z* values corresponding to important metabolites, the list of metabolites was arranged by two databases: the HMDB online database (available online: http://www.hmdb.ca) and MI-Pack software [31]. The results are summarized in Table S2 and some overlap was observed between these two databases.

### 3.5. Chemometrics and Statistical Analyses

Before principal component analysis (PCA), data set normalization was performed based on the probabilistic quotient normalization (PQN) method [33] to reduce the effect of extreme peak intensities. Next, the data matrix was treated using the K-nearest neighbor (KNN) imputation technique [34,35] to estimate the missing values. Finally, the samples were transformed using the generalized log transformation (GLog) method [36] to remove the domination of the highest intensity peaks through stabilizing the whole variance. PCA was then performed using MATLAB software and PLS Toolbox, which is an unsupervised approach, and the obtained information represents any structure correlated to the data. Indeed, PCA converts data into a simple visual format. The PCA score plot is based on similarities or differences in each sample, which are due to their concentrations or compositions. Each mass spectrum in score plot is converted to one point in the space of principal components. In PLSDA, a supervised method, the loading plot represents the relative contribution of each *m*/*z* value, corresponding to one metabolite, to the principal components. Therefore, it shows *m*/*z* values of metabolites responsible for discrimination among classes in score plot. The identification of important metabolites depends on determining the latent variables responsible for class separation.

Different statistical methods are available in order to determine metabolites that are significantly different from metabolites present in the center of the loading plot. The different *m*/*z* values from loading plot were calculated according to the formula:

υ − ῡ ≥ α × σ(1)

where υ is loading matrix; ῡ is average of loading matrix; σ is standard deviation of loading matrix; and α is dependent on the percent confident that the analysis is correct. In the present study, α was selected equal to two.

However, data visualization plays a pivotal role in the interpretation of metabolomics results. In a heat map, columns and rows are rearranged to find a quantitative pattern from the considered data. Then, these rearranged data are converted to a color image. This type of data visualization is very informative [33].

## 4. Conclusions

Investigations with respect to the impact of different abiotic and biotic stresses (such as water deficit) on relevant food and medicinal plants are very informative and useful, and will undoubtedly help plant producers and breeders to improve their knowledge on the tolerance mechanisms of these plants against stresses. This will assist them in gaining more yield under stressed conditions. In this view, we evaluated, for the first time, lipidome changes induced by drought stress in sensitive and tolerant thyme plants. Our results showed significant changes of various lipid classes in leaves of plants exposed to severe water deficiency. In particular, total lipids and galactolipids (MGDGs and DGDGs) as well as phospholipids (PG, PE, PA and PS) decreased in stressed sensitive plants. Conversely, in tolerant plants, severe drought increased galactolipids and unsaturated fatty acids (18:2 and 18:3) in addition to antioxidant secondary metabolites (i.e., flavonoids).

Therefore, we can speculate that water deficiency stimulates the biosynthesis of membrane structural lipids in tolerant plants in order to protect cell and chloroplast membranes from damage induced by severe drought and preserve their structure and function. In addition, increased levels of antioxidant lipids and flavonoids, in these plants, would contribute to scavenging ROS production during water deficiency.

Finally, in the current global climate change scenario, further molecular approaches, such as transcriptomics and RNAseq, will certainly contribute to understanding the gene network behind the observed phenomena, thus providing a new insight into plant adaptation mechanisms with respect to drought stress conditions.

## Figures and Tables

**Table 1:** Major metabolic pathways significantly involved in drought stress response of thyme plants.

Pathway	Category	Coverage	*p*-Value	Database
α-Linolenic acid metabolism	Lipid metabolism	0.33	0.0068255	MI-Pack, MetaboAnalyst
Isoflavonoid biosynthesis	Biosynthesis of secondary metabolites	0.25	n/a	MI-Pack
Linoleic acid metabolism	Lipid metabolism	0.23	n/a	MI-Pack
Limonene and pinene degradations	Metabolism of terpenoids and polyketides	0.2	0.021087	MetaboAnalyst
Retinol metabolism	Metabolism of cofactors and vitamins	0.13	n/a	MI-Pack
Biosynthesis of unsaturated fatty acids	Lipid metabolism	0.12	0.025999	MetaboAnalyst
Steroid biosynthesis	Lipid metabolism	0.1	n/a	MI-Pack
Flavonoid biosynthesis	Biosynthesis of secondary metabolites	0.1	n/a	MI-Pack

## Supplementary Materials

Supplementary materials can be found at www.mdpi.com/1422-0067/18/10/2067/s1.

## Abbreviations

DGDG	Digalactosyldiacylglycerol
DI FT-ICR	Direct infusion Fourier transform ion cyclotron resonance
FFA	Free fatty acid
KNN	K-nearest neighbor imputation method
MGDG	Monogalactosyldiacylglycerol
NL	Neutral lipids
PA	Phosphatidic acid
PC	Phosphatidylcholine
PCA	Principal component analysis
PE	Phosphatidylethanolamine
PG	Phosphatidylglycerol
PI	Phosphatidylinositol
PL	Phospholipids
PLS	Partial least square
PLSDA	Partial least square discriminant analysis
PQN	Probabilistic quotient normalization
PS	Phosphatidylserine
PUFAs	Polyunsaturated fatty acids
ROS	Reactive oxygen species
VIP	Variable importance on projection
18:3, 18:2	18-Carbon chain PUFAs with three and two double bonds, respectively

## References

[1] Reddy A.R., Chaitanya K.V., Vivekanandan M. (2004). Drought-induced responses of photosynthesis and antioxidant metabolism in higher plants. J. Plant Physiol..

[2] Seki M., Umezawa T., Urano K., Shinozaki K. (2007). Regulatory metabolic networks in drought stress responses. Curr. Opin. Plant Biol..

[3] Miller G., Shulaev V., Mittler R. (2008). Reactive oxygen signaling and abiotic stress. Physiol. Plant..

[4] Stahl-Biskup E., Sàez F. (2002). Thyme: The Genus Thymtls.

[5] Balentine D.A., Albano M.C., Nair M.G. (1999). Role of medicinal plants, herbs, and spices in protecting human health. Nutr. Rev..

[6] Caragay A.B. (1992). Cancer-preventive foods and ingredients. Food Technol..

[7] Craig W.J. (1999). Health-promoting properties of common herbs. Am. J. Clin. Nutr..

[8] Zaika L.L. (1988). Spices and herbs: Their antimicrobial activity and its determination1. J. Food Saf..

[9] Aziz A., Larher F. (1998). Osmotic stress induced changes in lipid composition and peroxidation in leaf discs of *Brassica napus* L.. J. Plant Physiol..

[10] Dakhma W.S., Zarrouk M., Cherif A. (1995). Effects of drought-stress on lipids in rape leaves. Phytochemistry.

[11] Svenningsson H., Liljenberg C. (1986). Membrane lipid changes in root cells of rape (*brassicanapus*) as a function of water-deficit stress. Physiol. Plant..

[12] Quartacci M.F., Pinzino C., Sgherri C.L., Navari-Izzo F. (1995). Lipid composition and protein dynamics in thylakoids of two wheat cultivars differently sensitive to drought. Plant Physiol..

[13] Hubac C., Guerrier D., Ferran J., Tremolieres A. (1989). Change of leaf lipid composition during water stress in two genotypes of lupinus albus resistant or susceptible to drought. Plant Physiol. Biochem..

[14] Bahl J., Francke B., Monéger R. (1976). Lipid composition of envelopes, prolamellar bodies and other plastid membranes in etiolated, green and greening wheat leaves. Planta.

[15] Eastman P., Rashid A., Camm E. (1998). Changes of the photosystem 2 activity and thylakoid proteins in spruce seedlings during water stress. Photosynthetica.

[16] Pham T.A.T., Vieira da Silva J., Mazliak P. (1990). The role of membrane lipids in drought resistance of plants. Bulletin de la Société Botanique de France. Actualités Botaniques.

[17] Kaoua M., Serraj R., Benichou M., Hsissou D. (2006). Comparative sensitivity of two moroccan wheat varieties to water stress: The relationship between fatty acids and proline accumulation. Bot. Stud..

[18] El-Hafid L., Pham T.A., Zuily-Fodil Y., Vieira da Silva J. Enzymatic Breakdown of Polar Lipids in Cotton Leaves under Water Stress. 1. Degradation of Monogalactosyl-Diacylglycerol. Plant Physiology Biochemistry 1989. http://agris.fao.org/agris-search/search.do?recordID=FR9001726.

[19] Anh T.P.T., Borrel-Flood C., da Silva J.V., Justin A.M., Mazliak P. (1985). Effects of water stress on lipid metabolism in cotton leaves. Phytochemistry.

[20] Hamrouni I., Salah H.B., Marzouk B. (2001). Effects of water-deficit on lipids of safflower aerial parts. Phytochemistry.

[21] De Paula F.M., Thi A.P., de Silva J.V., Justin A., Demandre C., Mazliak P. (1990). Effects of water stress on the molecular species composition of polar lipids from *Vigna unguiculata* L. Leaves. Plant Sci..

[22] Repellin A., Thi A.P., Tashakorie A., Sahsah Y., Daniel C., Zuily-Fodil Y. (1997). Leaf membrane lipids and drought tolerance in young coconut palms (*Cocos nucifera* L.). Eur. J. Agron..

[23] Gigon A., Matos A.-R., Laffray D., Zuily-Fodil Y., Pham-Thi A.T. (2004). Effect of drought stress on lipid metabolism in the leaves of *Arabidopsis thaliana* (ecotype columbia). Ann. Bot..

[24] Upchurch R.G. (2008). Fatty acid unsaturation, mobilization, and regulation in the response of plants to stress. Biotechnol. Lett..

[25] Ramadan A., Sabir J.S., Alakilli S.Y., Shokry A.M., Gadalla N.O., Edris S., Al-Korduy M.A., Al-Zahrani H.S., El-Domyati F.M., Bahieldin A. (2014). Metabolomic response of calotropis procera growing in the desert to changes in water availability. PLoS ONE.

[26] Okazaki Y., Saito K. (2014). Roles of lipids as signaling molecules and mitigators during stress response in plants. Plant J..

[27] Moradi P. (2014). Use of Metabolomics to Study Water Deficit Stress on the Medicinal Plant Thyme. Ph.D. Thesis.

[28] Moellering E.R., Muthan B., Benning C. (2010). Freezing tolerance in plants requires lipid remodeling at the outer chloroplast membrane. Science.

[29] Perez-Enciso M., Tenenhaus M. (2003). Prediction of clinical outcome with microarray data: A partial least squares discriminant analysis (pls-da) approach. Hum. Genet..

[30] Wold S., Trygg J., Berglund A., Antti H. (2001). Some recent developments in pls modeling. Chemometr. Intell. Lab. Syst..

[31] Gromski P.S., Muhamadali H., Ellis D.I., Xu Y., Correa E., Turner M.L., Goodacre R. (2015). A tutorial review: Metabolomics and partial least squares-discriminant analysis—A marriage of convenience or a shotgun wedding. Anal. Chim. Acta.

[32] Dettmer K., Aronov P.A., Hammock B.D. (2007). Mass spectrometry-based metabolomics. Mass Spectrom. Rev..

[33] Key M. (2012). A tutorial in displaying mass spectrometry-based proteomic data using heat maps. BMC Bioinform..

[34] Moon J.-Y., Jung H.-J., Moon M.H., Chung B.C., Choi M.H. (2009). Heat-map visualization of gas chromatography-mass spectrometry based quantitative signatures on steroid metabolism. J. Am. Soc. Mass Spectrom..

[35] Lenka S.K., Katiyar A., Chinnusamy V., Bansal K.C. (2011). Comparative analysis of drought-responsive transcriptome in indica rice genotypes with contrasting drought tolerance. Plant Biotechnol. J..

[36] Cho S.-M., Park J.-Y., Han S.-H., Anderson A.J., Yang K.-Y., Gardener B.M., Kim Y.-C. (2011). Identification and transcriptional analysis of priming genes in arabidopsis thaliana induced by root colonization with pseudomonas chlororaphis o6. Plant Pathol. J..

[37] Song L., Prince S., Valliyodan B., Joshi T., dos Santos J.V.M., Wang J., Lin L., Wan J., Wang Y., Xu D. (2016). Genome-wide transcriptome analysis of soybean primary root under varying water-deficit conditions. BMC Genom..

[38] García-Calderon M., Pons-Ferrer T., Mrázova A., Pal‘ove-Balang P., Vilková M., Pérez-Delgado C.M., Vega J.M., Eliášová A., Repčák M., Márquez A.J. (2015). Modulation of phenolic metabolism under stress conditions in a lotus japonicus mutant lacking plastidic glutamine synthetase. Front. Plant Sci..

[39] Nakabayashi R., Yonekura-Sakakidara K., Urano K., Suzuki M., Yamada Y., Nishizawa T., Matsuda F., Kojima M., Sakakibara H., Shinozaki K. (2014). Enhancement of oxidative and drought tolerance in arabidopsis by overaccumulation of antioxidant flavonoids. Plant J..

[40] Shojaie B., Mostajeran A., Ghanadian M. (2016). Flavonoid dynamic responses to different drought conditions: Amount, type, and localization of flavonols in roots and shoots of *Arabidopsis thaliana* L.. Turk. J. Biol..

