# Supplementary material for: Lipidomics Unravels the Role of Leaf Lipids in Thyme Plant Response to Drought Stress
Source: Int J Mol Sci. 2017 Sep 28;18(10):2067. doi: 10.3390/ijms18102067 (PMC5666749; doi:10.3390/ijms18102067)
Supplement: Supplementary file 1 [file ijms-18-02067-s001.zip › ijms-218416-Supplementary materials/fig. s1.pdf]

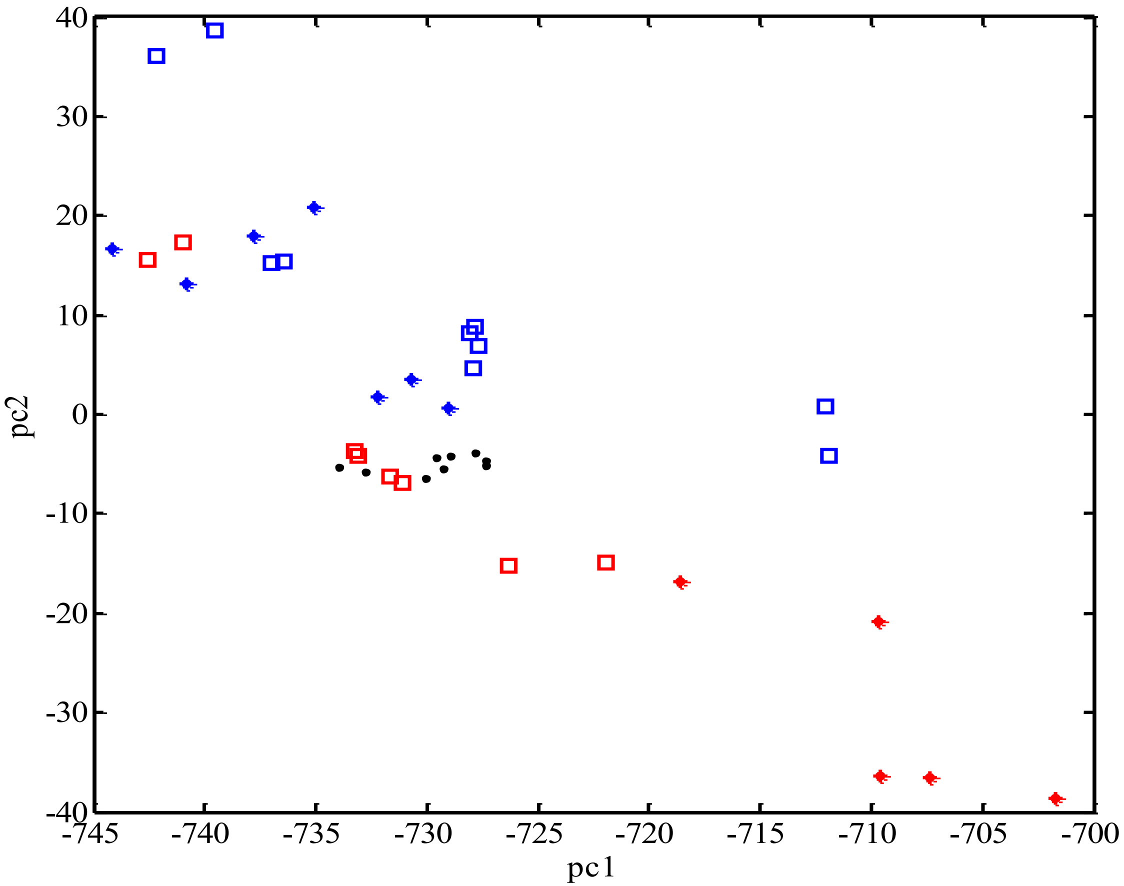

Figure S1. Principal component analysis on lipidomics dataset of thyme plants exposed to drought stress. ★ Tolerant, watered (TW); ▯ tolerant, drought (TD); ★ sensitive, watered (SW), ▫ sensitve, drought (SD); ⬮ quality control (QC).
