# Supplementary material for: Lipidomics Unravels the Role of Leaf Lipids in Thyme Plant Response to Drought Stress
Source: Int J Mol Sci. 2017 Sep 28;18(10):2067. doi: 10.3390/ijms18102067 (PMC5666749; doi:10.3390/ijms18102067)
Supplement: Supplementary file 1 [file ijms-18-02067-s001.zip › ijms-218416-Supplementary materials/Table S1.docx]

Table S1. Full list of identified metabolites significantly changed in sensitive plants under water deficit condition.

| **m/z** | **watered** | **Droughted** | **Fold change** | **p_value** | **Metabolite name** |
| --- | --- | --- | --- | --- | --- |
| 125.0357 | 3122.75664 | 1931.222 | 0.618435 | 0.0478 | Thymine |
| 137.0245 | 175879.6 | 279732.2 | 1.590475 | 0.011472 | 3,4-Dihydroxybenzaldehyde, 3-Hydroxybenzoate |
| 144.0456 | 2363.78 | 10566.84 | 4.470315 | 0.007611 | 8-Hydroxyquinoline, Indole-3-carboxaldehyde |
| 151.0402 | 16947.57 | 41138.22 | 2.427382 | 0.019944 | 4-Hydroxy-3-methoxy-benzaldehyde, Methyl salicylate, vanillin |
| 195.03 | 25590.75 | 40201.57 | 1.570941 | 0.048558 | 3,4,6-trihydroxy-cis-cinnamate, 3-(2-propenoic acid)-4,6-hydroxy cyclohexa-2,5-dienone, 3-(3,4-Dihydroxyphenyl)pyruvate |
| 225.186 | 31607.32 | 20285.65 | 0.641802 | 0.011224 | Myristoleate, myristate |
| 227.0925 | 59410.12 | 97535.83 | 1.641738 | 0.006305 | 1,3,5-Trimethoxybenzene (JAN) |
| 229.0274 | 19934.78 | 56814.33 | 2.850011 | 0.02786 | 5-Hydroxyconiferaldehyde , ferulic acid |
| 281.2486 | 2808600 | 1817895 | 0.64726 | 0.038825 | (6Z)-Octadecenoic acid , (9Z)-Octadecenoic acid , cis-2-octadecenoate, octadecadiene-1,18-diol, stearate |
| 293.2487 | 19099.48 | 15763.02 | 0.825311 | 0.015758 | Sterculic acid |
| 309.2072 | 191441.6 | 309367.2 | 1.615987 | 0.021926 | 13(S)-hydroperoxylinolenate, 13S-HpOTrE, 2-R-hydroperoxy-linolenate, 6,9-octadecadienedioate |
| 311.0562 | 11847.41 | 23687.45 | 1.999378 | 0.010442 | 2-carboxyanthraquinone |
| 313.0616 | 5380.02 | 7155.098 | 1.329939 | 0.038502 | Geranyl diphosphate |
| 319.2224 | 10857.35 | 5646.377 | 0.520051 | 0.002864 | (6Z)-Octadecenoic acid, (9Z)-Octadecenoic acid , cis-2-octadecenoate, octadecadiene-1,18-diol |
| 327.2905 | 20234.74 | 44815.95 | 2.214802 | 0.000164 | 2-hydroxy-eicosanoate, 20-hydroxyeicosanoate |
| 331.0824 | 30076.72 | 72740.83 | 2.41851 | 0.020842 | Pinobanksin, licodione, naringenin, naringenin chalcone |
| 331.1551 | 162101 | 58525.08 | 0.361041 | 0.022945 | Gibberellin A20, Gibberellin A4, Gibberellin A51 |
| 356.2807 | 12341.92 | 7177.372 | 0.581544 | 0.01485 | 4,8-sphingadienine |
| 357.2072 | 9952.18 | 6546.91 | 0.657837 | 0.042633 | Δ-9-tetrahydrocannabinolic acid |
| 373.2597 | 12143.86 | 9208.202 | 0.75826 | 0.009809 | 9,10-epoxystearate |
| 379.2493 | 13613.8 | 18522.48 | 1.360567 | 0.008125 | sphinganine 1-phosphate |
| 383.3532 | 40514.32 | 64362.5 | 1.588636 | 0.013338 | 24-hydroxytetracosanoate, DL-Cerebronic acid |
| 387.1298 | 5096.452 | 9064.102 | 1.778512 | 0.03454 | Secologanin |
| 395.075 | 6368.138 | 8809.118 | 1.383311 | 0.003692 | Chrysosplenetin |
| 407.2595 | 58763.64 | 37071.92 | 0.630865 | 0.044034 | 3,5-dihydroxy-6,7-didehydro-12-apo-&beta;-caroten-12-al, 5,6-epoxy-3-hydroxy-12-apo-&beta;-caroten-12-al |
| 414.2944 | 173079.8 | 46985.6 | 0.271468 | 0.001308 | β-apo-8-carotenal |
| 423.4209 | 51972.3 | 104959.8 | 2.019534 | 0.000647 | Octacosanoic acid |
| 429.374 | 19947 | 10461.54 | 0.524467 | 0.001441 | 22α-hydroxy-sitosterol, 4alpha-hydroxymethyl-4beta-methyl-5alpha-cholesta-8-en-3beta-ol, 4beta-(hydroxymethyl)-4alpha-methyl-5alpha-cholest-7-en-3β-ol, alpha-Tocopherol |
| 437.4371 | 16754.66 | 27850.37 | 1.662246 | 7.86E-05 | Nonacosanoic acid, heptacosanoate, 25-methyl-methyl ester, 4-heptacosene, 6-heptacosene |
| 441.2652 | 17671.96 | 9417.78 | 0.532922 | 0.000393 | 3S,5R,6S-5,6-epoxy-3-hydroxy-5,6-dihydro-12-apo-&beta;-caroten-12-al |
| 449.2551 | 22361.24 | 15919.6 | 0.711928 | 0.025997 | 1-16:1-lysoPE |
| 449.2915 | 12274.57 | 17203.25 | 1.401536 | 0.031002 | 1,2-benzenedicarboxylate acid, diisooctyl ester |
| 451.4527 | 111281 | 180178.8 | 1.619134 | 0.000991 | Melissic acid |
| 453.338 | 11366.92 | 37006.02 | 3.255588 | 0.000149 | 5,7,22,24(28)-ergostatetraenol |
| 455.3172 | 11675.13 | 26522.23 | 2.271686 | 0.001153 | 2-methyl-6-geranylgeranyl-1,4-benzoquinol |
| 455.3527 | 7445538 | 21121750 | 2.836833 | 0.002145 | 3-keto-4-methylzymosterol, 5,7,24(28)-ergostatrienol, 5-dehydro episterol |
| 465.3228 | 13367.36 | 17164.7 | 1.284076 | 0.044735 | 28-norbrassinolide |
| 465.4683 | 26499.64 | 43721.08 | 1.649875 | 0.004847 | 4-nonacosene, 6-nonacosene |
| 467.2805 | 60564.08 | 37767.33 | 0.623593 | 0.030855 | 3,5-dihydroxy-6,7-didehydro-12-apo-&beta;-caroten-12-al, 5,,6-epoxy-3-hydroxy-12-apo-&beta;-caroten-12-al |
| 471.3485 | 203394.6 | 2167474 | 10.65649 | 0.01461 | 4α-formyl-5α-cholesta-8,24-dien-3β-ol, 2-hydroxyoleanolate |
| 473.3642 | 17615.18 | 42271.83 | 2.399739 | 2.38E-05 | 22α-hydroxy-campest-4-en-3-one, 4Alpha-hydroxymethyl-5alpha-cholesta-8,24-dien-3beta-ol , 4alpha-formyl-5alpha-cholesta-8-en-3beta-ol |
| 475.4163 | 16054.54 | 24767.2 | 1.542691 | 0.016467 | sitostanol |
| 479.484 | 155592.2 | 251311.5 | 1.615193 | 0.025574 | Lacceroic acid |
| 487.3436 | 52654 | 451330 | 8.571618 | 0.001077 | 4α-carboxy-5α-cholesta-8,24-dien-3β-ol |
| 493.4997 | 57140.14 | 81736.37 | 1.430454 | 0.047129 | 4-hentriacontene, 6-hentriacontene, Psyllic acid |
| 505.3536 | 6890.696 | 9959.795 | 1.445398 | 0.01092 | 3-dehydroteasterone |
| 519.3331 | 6711.288 | 13783.15 | 2.053726 | 6.64E-05 | 1-Linoleoylglycerophosphocholine |
| 521.3488 | 9868.386 | 15372.68 | 1.557771 | 0.001486 | 1-Oleoylglycerophosphocholine |
| 528.2689 | 4398.192 | 7550.793 | 1.716795 | 0.004067 | 1-18:2-lysoPE |
| 531.3696 | 23063.6 | 37240.65 | 1.614694 | 0.022813 | 2-hydroxyoleanolate |
| 537.3799 | 14605.23 | 20234.3 | 1.385415 | 0.010343 | (22R,23R)-28-homocastasterone |
| 550.4848 | 8592.794 | 4184.098 | 0.486931 | 0.014303 | N-(2-hydroxyhexadecanoyl)-4,8-sphingadienine |
| 553.3751 | 7229.77 | 9987.982 | 1.381508 | 0.000397 | 28-homobrassinolide |
| 555.2241 | 6372.042 | 12406.72 | 1.947055 | 0.005108 | all-trans-Pentaprenyl diphosphate |
| 563.39 | 45515.12 | 26478.6 | 0.581754 | 0.040648 | 3-hydroxy-4-ketotorulene, Canthaxanthin |
| 581.4009 | 91686.26 | 35552.62 | 0.387764 | 0.001258 | 4-ketolutein |
| 599.4114 | 175734 | 83694.87 | 0.476259 | 0.006062 | 9-cis-violaxanthin, 9-cis-Neoxanthin, Neoxanthin, Violaxanthin |
| 607.292 | 20135.29 | 59764.52 | 2.968147 | 0.048568 | Presqualene diphosphate, all-trans-Hexaprenyl diphosphate |
| 659.4329 | 1186391 | 738191.5 | 0.622216 | 0.033141 | 9-cis-violaxanthin, 9-cis-Neoxanthin, Neoxanthin, Violaxanthin |
| 719.4881 | 772442 | 392665.3 | 0.508343 | 0.007946 | 16:0-t16:1-PG, 1,2-dipalmitoyl-phosphatidylglycerol |
| 723.4256 | 29781.92 | 44638.07 | 1.498831 | 0.020108 | 18:3-16:3-PA |
| 738.5097 | 141906.7 | 57158.47 | 0.402789 | 0.002177 | 18:1-18:3-PE |
| 741.4721 | 2142180 | 1320164 | 0.616271 | 0.046998 | 18:3-t16:1-PG |
| 742.476 | 912712.8 | 558991.7 | 0.612451 | 0.045539 | 1,2-dipalmitoyl-phosphatidylglycerol |
| 742.5406 | 33131.66 | 14350.44 | 0.433134 | 0.036355 | 18:0-18:2-PE |
| 745.5041 | 923812 | 508771.5 | 0.550731 | 0.041668 | 18:1-t16:1-PG, 18:2-16:0-PG |
| 751.5381 | 183447 | 68812.72 | 0.37511 | 5.88E-05 | 18:0-16:3-MGDG, 18:1-16:2-MGDG, 18:2-16:1-MGDG, 18:3-16:0-MGDG |
| 755.4754 | 88418.5 | 51437.68 | 0.581752 | 0.01707 | 16:0-18:3-PS |
| 790.5221 | 1251113 | 874365.2 | 0.69887 | 0.045534 | 18:2-18:3-MGDG, 18:3-18:2-MGDG |
| 802.4649 | 86220.08 | 56311.3 | 0.653111 | 0.038749 | 18:2-18:3-PS |
| 815.5279 | 21781.74 | 13415.16 | 0.61589 | 0.035435 | 18:1-18:3-MGDG, 18:2-18:2-MGDG |
| 817.5419 | 9019.24 | 5160.898 | 0.57221 | 0.002539 | 18:0-18:3-MGDG, 18:1-18:2-MGDG |
| 819.5557 | 16730.4 | 8530.533 | 0.509882 | 0.002335 | 18:0-18:3-PC, 18:1-18:2-PC, 18:2-18:1-PC, 18:0-18:2-MGDG, 18:1-18:1-MGDG |
| 820.5335 | 1481526 | 815470.8 | 0.550426 | 0.009297 | 1-Hexadecanoyl-2-(9Z-octadecenoyl)-sn-glycero-8-phosphoserine |
| 840.5053 | 314830.8 | 84746.6 | 0.269181 | 0.010424 | 18:2-18:3-PS |
| 842.5207 | 330417 | 115240.5 | 0.348773 | 0.008365 | 18:2-18:2-PS |
| 845.5516 | 60542.18 | 27299.15 | 0.450911 | 0.02456 | 20:2-18:3-PC |
| 848.5681 | 23007.46 | 12703.14 | 0.552131 | 0.017143 | 18:0-18:1-PS |
| 935.5757 | 6622716 | 3880308 | 0.585909 | 0.01558 | 18:3-18:3-DGDG |
| 936.5793 | 3221806 | 1882470 | 0.58429 | 0.017801 | 18:2-18:3-DGDG, 18:3-18:2-DGDG |
| 971.5966 | 146426 | 78190.83 | 0.533996 | 3.98E-05 | 18:2-16:2-DGDG, 18:3-16:1-DGDG |
| 973.6132 | 3814426 | 2049320 | 0.537255 | 0.003821 | 16:0-18:3-DGDG, 16:1-18:2-DGDG, 18:3-16:0-DGDG |
| 995.5966 | 2666850 | 683293.3 | 0.256217 | 0.000371 | 18:3-18:3-DGDG |
