# Supplementary material for: Lipidomics Unravels the Role of Leaf Lipids in Thyme Plant Response to Drought Stress
Source: Int J Mol Sci. 2017 Sep 28;18(10):2067. doi: 10.3390/ijms18102067 (PMC5666749; doi:10.3390/ijms18102067)
Supplement: Supplementary file 1 [file ijms-18-02067-s001.zip › ijms-218416-Supplementary materials/Table S2.docx]

Table S2. Full list of identified metabolites significantly changed in tolerant plants under water deficit condition.

| **m/z** | **watered** | **Droughted** | **Fold change** | **p_value** | **Metabolite name** |
| --- | --- | --- | --- | --- | --- |
| 227.0925 | 45123.5 | 92371.6 | 2.047084 | 0.01218 | 1,3,5-Trimethoxybenzene (JAN) |
| 271.1705 | 8594.666 | 6102.842 | 0.710073 | 0.047346 | estradiol |
| 291.1967 | 285580.6 | 142493 | 0.498959 | 0.037567 | (9Z)-(13S)-12,13-epoxyoctadeca-9,11-dienoate, 12,13(S)-epoxylinolenate, 12-oxo-cis-10,15-phytodienoate |
| 295.2279 | 111585.4 | 47477.4 | 0.42548 | 0.004673 | 16-oxo-palmitate, 18-hydroxyoctadeca-9Z,12Z-dienoate, vernoleate |
| 297.1861 | 148886.6 | 116494.4 | 0.782437 | 0.035449 | retinoate |
| 309.2072 | 436312.2 | 269937.2 | 0.618679 | 0.029825 | 13(S)-hydroperoxylinolenate, 13S-HpOTrE, 2-R-hydroperoxy-linolenate, 6,9-octadecadienedioate |
| 311.2229 | 129411.1 | 52809.2 | 0.408073 | 0.007721 | 13(S)-hydroperoxyoctadeca-9,11-dienoate, hexadecanedioate |
| 327.2905 | 127945.3 | 21843.36 | 0.170724 | 0.009202 | 2-hydroxy-eicosanoate, 20-hydroxyeicosanoate, Octadecanal |
| 331.0824 | 16443 | 32121.18 | 1.953487 | 0.014556 | Pinobanksin, licodione, naringenin, naringenin chalcone |
| 331.1551 | 49478.8 | 89202.2 | 1.802837 | 0.026517 | Gibberellin A_20_, Gibberellin A_4_, Gibberellin A_51_ |
| 347.0773 | 47995.92 | 90774.9 | 1.891305 | 0.018068 | dihydrokaempferol, eriodictyol |
| 347.1864 | 9066.04 | 21685.62 | 2.391962 | 0.006483 | GA_110_, Gibberellin A_14_, Gibberellin A_15_ open lactone, Gibberellin A_53_ |
| 417.3222 | 1650804 | 2466114 | 1.493887 | 0.017791 | 1-monostearin |
| 419.3294 | 35622.18 | 51043.94 | 1.432926 | 0.021547 | 24-methyldesmosterol, Campest-4-en-3-one, brassicasterol, crinosterol, episterol, Pentacosanoic acid, 24-hydroxytetracosanoate, DL-Cerebronic acid |
| 429.374 | 10292.61 | 22443.58 | 2.180552 | 2.70E-05 | 22α-hydroxy-sitosterol, 4alpha-hydroxymethyl-4beta-methyl-5alpha-cholesta-8-en-3beta-ol, 4beta-(hydroxymethyl)-4alpha-methyl-5alpha-cholest-7-en-3beta-ol, α-Tocopherol |
| 445.3246 | 9454.32 | 15112.74 | 1.598501 | 0.00112 | 4α-methyl-5α-ergosta-8,14,24(28)-trien-3&beta;-ol, 4,4-dimethyl-5-α-cholesta-8,14,24-trien-3-&beta;-ol , -Dehydroavenasterol, 4,4-Diapophytoene |
| 489.3591 | 11692.45 | 7328.398 | 0.626763 | 0.005765 | 4α-carboxy-5-α-cholesta-8-en-3β-ol |
| 505.3536 | 12441.32 | 7310.812 | 0.587623 | 0.000142 | 3-dehydroteasterone |
| 509.3851 | 16916.44 | 13248.44 | 0.78317 | 0.006516 | 6-deoxocastasterone |
| 519.3331 | 18177.02 | 9564.566 | 0.52619 | 0.007652 | 1-Linoleoylglycerophosphocholine |
| 521.3488 | 15213.86 | 9653.548 | 0.634523 | 0.001961 | 1-Oleoylglycerophosphocholine |
| 523.3642 | 16943.44 | 14877.48 | 0.878067 | 0.016729 | 1-18:0-lysoPC, castasterone |
| 552.5003 | 16387.96 | 8409.272 | 0.513137 | 0.004599 | 4-hydroxysphing-8(E)-enine-16:0, ceramide, 4-hydroxysphing-8(Z)-enine-16:0, ceramide |
| 559.2356 | 52789.83 | 11030.52 | 0.208952 | 0.012233 | protoporphyrin IX |
| 581.3709 | 14564.52 | 7412.89 | 0.508969 | 1.39E-05 | 1-Oleoylglycerophosphocholine |
| 581.4009 | 26686.38 | 37276.54 | 1.396838 | 0.016164 | 4-ketolutein |
| 599.4114 | 58569.04 | 78097.82 | 1.333432 | 0.027119 | 9-*cis*-violaxanthin , 9-*cis*-Neoxanthin , Neoxanthin, Violaxanthin |
| 623.2868 | 17793.46 | 7942.556 | 0.446375 | 0.012151 | Presqualene diphosphate, all-trans-Hexaprenyl diphosphate |
| 636.5944 | 19099.28 | 12791.36 | 0.66973 | 0.002439 | 4-hydroxysphing-8(E)-enine-22:0, ceramide, 4-hydroxysphing-8(Z)-enine-22:0, ceramide |
| 662.6107 | 7441.712 | 5325.092 | 0.715574 | 0.00795 | N-(2-hydroxytetracosanoyl)-4,8-sphingadienine |
| 692.6577 | 14371.58 | 9605.452 | 0.668364 | 0.000426 | 4-hydroxysphing-8(E)-enine-26:0, ceramide, 4-hydroxysphing-8(Z)-enine-26:0, ceramide |
| 696.6162 | 46949.42 | 20093.02 | 0.427972 | 0.005757 | 4-hydroxysphing-8(E)-enine-22:0, ceramide, 4-hydroxysphing-8(Z)-enine-22:0, ceramide |
| 697.4829 | 548727.2 | 785278.4 | 1.431091 | 0.048417 | 18:0-18:3-PA, 18:1-18:2-PA |
| 747.6096 | 52743.8 | 94409.74 | 1.789968 | 0.028254 | plastoquinone-9 |
| 751.5381 | 72862.88 | 105937.1 | 1.453924 | 0.004963 | 18:0-16:3-MGDG, 18:1-16:2-MGDG , 18:2-16:1-MGDG, 18:3-16:0-MGDG |
| 752.679 | 16650.02 | 9465.278 | 0.568484 | 0.000863 | 4-hydroxysphing-8(E)-enine-26:0, ceramide, 4-hydroxysphing-8(Z)-enine-26:0, ceramide |
| 791.5746 | 64026.96 | 90811.16 | 1.418327 | 0.008214 | 2-nonaprenyl-6-methoxy-1,4-benzoquinol |
| 792.5774 | 17411.36 | 24176.64 | 1.388556 | 0.021756 | 1,2-dipalmitoyl-phosphatidylcholine |
| 802.4649 | 76863.28 | 156135.7 | 2.031343 | 0.02901 | 18:2-18:3-PS |
| 816.5037 | 2942034 | 4236122 | 1.439862 | 0.006599 | 16:0-18:3-PS |
| 817.5419 | 7503.368 | 13167.31 | 1.754854 | 0.00745 | 18:0-18:3-MGDG , 18:1-18:2-MGDG |
| 819.5557 | 13019.98 | 19931.18 | 1.530815 | 0.001727 | 18:0-18:3-PC, 18:1-18:2-PC, 18:2-18:1-PC, 18:0-18:2-MGDG, 18:1-18:1-MGDG, 18:1-18:3-MGDG, 18:2-18:2-MGDG |
| 820.5335 | 861404.4 | 1328790 | 1.542586 | 0.001153 | 1-Hexadecanoyl-2-(9Z-octadecenoyl)-sn-glycero-3-phosphoserine |
| 832.5106 | 570955.8 | 662259.8 | 1.159914 | 0.048879 | 16:0-18:2-PI, 20:1-18:3-PS, 20:2-18:2-PS |
| 840.5053 | 257555.2 | 550734.2 | 2.138315 | 0.000372 | 18:2-18:3-PS |
| 842.5207 | 345632.4 | 532269.8 | 1.539988 | 0.027269 | 18:2-18:2-PS |
| 844.5362 | 279976.4 | 439478.8 | 1.569699 | 0.001309 | 18:0-18:3-PS, 18:1-18:2-PS |
| 845.5516 | 48197.96 | 111582.4 | 2.315085 | 0.002148 | 20:2-18:3-PC |
| 936.5793 | 1980274 | 2594834 | 1.310341 | 0.041392 | 18:2-18:3-DGDG, 18:3-18:2-DGDG |
| 971.5966 | 116958.2 | 149707.4 | 1.280008 | 0.030809 | 18:2-16:2-DGDG, 18:3-16:1-DGDG |
| 973.6132 | 2413822 | 3335048 | 1.381646 | 0.003221 | 16:0-18:3-DGDG, 16:1-18:2-DGDG, 18:3-16:0-DGDG |
