# Supplementary material for: Lipidomics Unravels the Role of Leaf Lipids in Thyme Plant Response to Drought Stress
Source: Int J Mol Sci. 2017 Sep 28;18(10):2067. doi: 10.3390/ijms18102067 (PMC5666749; doi:10.3390/ijms18102067)
Supplement: Supplementary file 1 [file ijms-18-02067-s001.zip › ijms-218416-Supplementary materials/Table S3.docx]

Table S3. Full list of metabolic pathways obtained from KEGG and metaboanalyst .

| Map ID | Map name | Sub total | Total | Coverage |
| --- | --- | --- | --- | --- |
| ko00592 | alpha-Linolenic acid metabolism | 13 | 40 | 0.33 |
| ko00943 | Isoflavonoid biosynthesis | 16 | 63 | 0.25 |
| ko00591 | Linoleic acid metabolism | 6 | 26 | 0.23 |
| ko00830 | Retinol metabolism | 3 | 24 | 0.13 |
| ko00100 | Steroid biosynthesis | 5 | 50 | 0.1 |
| ko00941 | Flavonoid biosynthesis | 7 | 68 | 0.1 |
| ko01040 | Biosynthesis of unsaturated fatty acids | 5 | 54 | 0.09 |
| ko00903 | Limonene and pinene degradation | 5 | 64 | 0.08 |
| ko00902 | Monoterpenoid biosynthesis | 3 | 44 | 0.07 |
| ko00311 | Penicillin and cephalosporin biosynthesis | 1 | 18 | 0.06 |
| ko00944 | Flavone and flavonol biosynthesis | 2 | 33 | 0.06 |
| ko00522 | Biosynthesis of 12-, 14- and 16-membered macrolides | 3 | 74 | 0.04 |
| map01061 | Biosynthesis of phenylpropanoids | 4 | 97 | 0.04 |
| map01070 | Biosynthesis of plant hormones | 3 | 68 | 0.04 |
| ko00622 | Xylene degradation | 1 | 37 | 0.03 |
| map01060 | Biosynthesis of plant secondary metabolites | 4 | 141 | 0.03 |
| ko00061 | Fatty acid biosynthesis | 1 | 49 | 0.02 |
| ko00904 | Diterpenoid biosynthesis | 2 | 87 | 0.02 |
| ko01110 | Biosynthesis of secondary metabolites | 25 | 1222 | 0.02 |
| ko00140 | Steroid hormone biosynthesis | 1 | 99 | 0.01 |
| ko00590 | Arachidonic acid metabolism | 1 | 75 | 0.01 |
| ko01057 | Biosynthesis of type II polyketide products | 2 | 134 | 0.01 |
| ko01100 | Metabolic pathways | 13 | 1478 | 0.01 |
| ko01120 | Microbial metabolism in diverse environments | 1 | 828 | 0 |

| Pathway Name | Total | Hits | p | -log(p) | Holm p | FDR | Impact |
| --- | --- | --- | --- | --- | --- | --- | --- |
| [alpha-Linolenic acid metabolism](http://www.metaboanalyst.ca/faces/Secure/pathway/ResultView.xhtml) | 23 | 5 | 0.0068255 | 4.9871 | 0.59382 | 0.59382 | 0.555 |
| [Biosynthesis of unsaturated fatty acids](http://www.metaboanalyst.ca/faces/Secure/pathway/ResultView.xhtml) | 42 | 5 | 0.07652 | 2.5702 | 1 | 1 | 0 |
| [Limonene and pinene degradation](http://www.metaboanalyst.ca/faces/Secure/pathway/ResultView.xhtml) | 15 | 2 | 0.19848 | 1.6171 | 1 | 1 | 0 |
| [Flavonoid biosynthesis](http://www.metaboanalyst.ca/faces/Secure/pathway/ResultView.xhtml) | 43 | 4 | 0.20879 | 1.5664 | 1 | 1 | 0.11751 |
| [Diterpenoid biosynthesis](http://www.metaboanalyst.ca/faces/Secure/pathway/ResultView.xhtml) | 26 | 1 | 0.77444 | 0.25561 | 1 | 1 | 0.0985 |
| [Steroid biosynthesis](http://www.metaboanalyst.ca/faces/Secure/pathway/ResultView.xhtml) | 36 | 1 | 0.87386 | 0.13484 | 1 | 1 | 0.00823 |
| [Fatty acid biosynthesis](http://www.metaboanalyst.ca/faces/Secure/pathway/ResultView.xhtml) | 49 | 1 | 0.94117 | 0.060633 | 1 | 1 | 0 |
