# Supplementary figures and images for: Lipidomics Unravels the Role of Leaf Lipids in Thyme Plant Response to Drought Stress
Source: Int J Mol Sci. 2017 Sep 28;18(10):2067. doi: 10.3390/ijms18102067 (PMC5666749; doi:10.3390/ijms18102067)

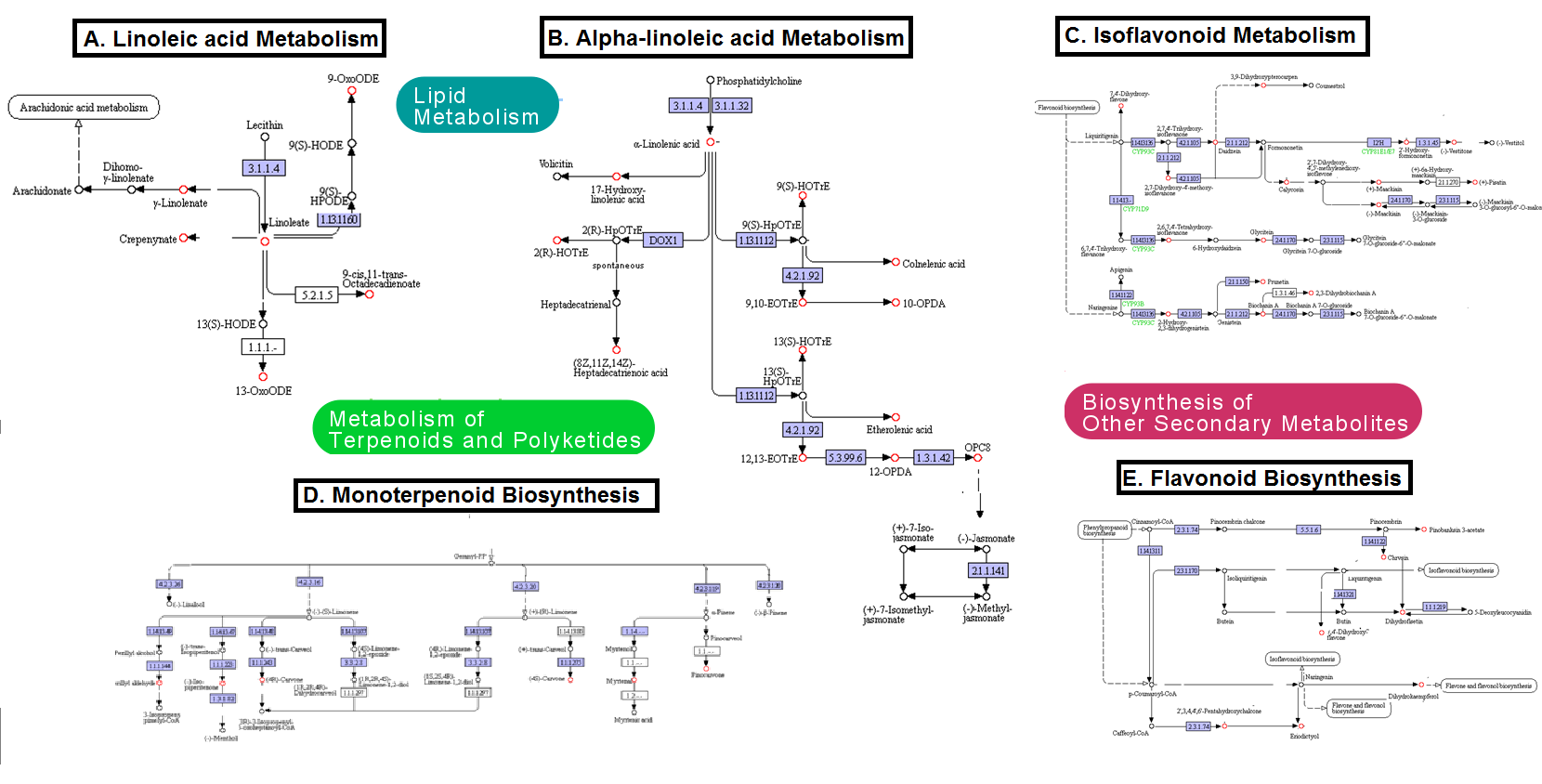

Supplement: Supplementary file 1 [file ijms-18-02067-s001.zip › ijms-218416-Supplementary materials/FigS2_total_map_highquality3.png]
